# Component-specific effects of PM_2.5_ on endoscopically identified erosive gastric mucosal lesions: a time-series study in Yixing, China

**DOI:** 10.3389/fpubh.2026.1891236

**Published:** 2026-08-11

**Authors:** Xuhua Mao, Yufei Yang, Xiaohong Wu, Qian Jiang, Han Sun

**Affiliations:** 1 Department of Clinical Laboratory, Innovation and Technology Transfer Center, Affiliated to Jiangsu University, Yixing, China; 2 Department of Clinical Laboratory, Precision Medicine Center, Affiliated to Jiangsu University, Yixing, China; 3 Department of Health Management Center, Yixing People’s Hospital, Affiliated to Jiangsu University, Yixing, China; 4 Department of General Surgery, Yixing People’s Hospital, Affiliated to Jiangsu University, Yixing, China; 5 Department of Gastroenterology, Xuzhou Central Hospital, Southeast University, Xuzhou, China

**Keywords:** air pollution, environmental epidemiology, erosive gastric mucosal lesions, Pm^2.5^ chemical components, time-series study

## Abstract

**Background:**

PM_2.5_ has been increasingly linked to digestive morbidity, but evidence on its short-term association with gastric mucosal injury remains limited. Few epidemiological studies have examined component-specific associations of PM_2.5_ with endoscopically identified erosive gastric mucosal lesions (EGML), common indicators of impaired mucosal defense.

**Methods:**

We consecutively enrolled individuals undergoing gastroscopy at Yixing People’s Hospital from January 1, 2024 to December 31, 2025, and collected their basic characteristics and gastroscopic findings. Daily grid concentrations of PM_2.5_ and its five chemical components were also extracted. We applied generalized additive models to explore the lag-specific correlations between particulate pollutants and endoscopically identified EGML. Attributable burden analysis was further performed to estimate the preventable number of cases under hypothetical reduction scenarios, and the relative contribution of each component was quantified.

**Results:**

A total of 60,789 individuals received gastroscopy, of whom 14,325 had EGML. A per interquartile range increase in PM_2.5_ concentration was associated with a 7.6% higher risk of EGML [incidence rate ratio (IRR): 1.076; 95% confidence interval (CI): 1.024, 1.132]. Among PM_2.5_ components, positive associations that remained significant after FDR correction were observed for sulfate (IRR: 1.070; 95% CI: 1.024, 1.118) and ammonium (IRR: 1.082; 95% CI: 1.026, 1.141). In contrast, no significant associations were found for nitrate (IRR: 1.012; 95% CI: 0.979, 1.046), organic matter (IRR: 1.012; 95% CI: 0.981, 1.043), and black carbon (IRR: 1.012; 95% CI: 0.982, 1.043).

**Conclusion:**

Short-term PM_2.5_ exposure is positively associated with EGML risk. Among the five components, sulfate and ammonium showed the strongest evidence of positive associations, although their independent contributions require further study. These findings extend existing epidemiological evidence on PM_2.5_ and gastric damage, and provide component-specific information that may inform targeted air quality management and population-level prevention of gastric mucosal diseases.

## Introduction

Erosive gastric mucosal lesions (EGML) are common findings in upper gastrointestinal endoscopy and are characterized by superficial mucosal defects confined to the muscularis mucosae without extension beyond it (1). Population-based estimates specifically for EGML remain limited; however, available endoscopy-based studies have reported that EGML-related conditions, including gastric erosions and erosive gastritis, are detected in approximately 10–30% of examined individuals, depending on the study population, endoscopic indication, medication exposure, and diagnostic criteria (2–4). These findings suggest that EGML-related mucosal injury is common in clinical and screening endoscopy settings and may impose a substantial healthcare burden. Although EGML are not equivalent to peptic ulcers, they indicate impaired gastric mucosal defense and may cause dyspeptic symptoms or upper gastrointestinal bleeding (5, 6). Established risk factors include *Helicobacter pylori* infection, alcohol consumption, nonsteroidal anti-inflammatory drug use, and so on (7–9). However, these factors do not fully explain short-term fluctuations in the detection of EGML. Given the high frequency and clinical relevance of EGML, identifying modifiable environmental triggers may help clarify preventable contributors to gastric mucosal injury.

The harmful influences of PM_2.5_ on respiratory and cardiovascular systems have been well documented (10, 11). Recent studies have revealed effects of PM_2.5_ on diverse digestive disorders, including inflammatory bowel disease and gastrointestinal hemorrhage (12, 13). The biological mechanisms underlying these associations likely involve systemic inflammation, oxidative stress, and gut microbiota dysbiosis triggered by inhaled particles (14, 15). Nevertheless, evidence remains limited regarding whether short-term PM_2.5_ exposure is associated with endoscopically identified EGML, and even less is known about the heterogeneous impacts of distinct PM_2.5_ chemical constituents. PM_2.5_ represents a complicated blend of chemical components, mainly including sulfate ( ${\mathrm{SO}}_{4}^{2-}$ ), nitrate ( ${\mathrm{NO}}_{3}^{-}$ ), ammonium ( ${\mathrm{NH}}_{4}^{+}$ ), organic matter (OM), and black carbon (BC) (16). These components originate from different emission sources and may exert distinct toxicological effects on human health (17). Understanding which specific components drive the gastric effects of PM_2.5_ is crucial for developing targeted air quality management strategies and identifying populations at higher risk of pollution-related mucosal injury.

To address these knowledge gaps, we collected data from over 60,000 individuals who underwent gastroscopy at a tertiary general hospital in eastern China. We examined the associations between daily concentrations of PM_2.5_ and its five major chemical components and the daily incidence of endoscopically identified EGML. By identifying PM_2.5_ constituents most strongly associated with EGML and quantifying their attributable burden, this study aims to provide evidence for targeted air pollution control and gastric mucosal injury prevention.

## Methods

### Ethics approval

This retrospective observational study was approved by the Ethics Committee of Yixing People’s Hospital (2026–059-01). The study period, January 1, 2024 to December 31, 2025, refers to the dates on which gastroscopic examinations were performed. The research-related extraction of electronic medical record data was conducted after ethics approval had been obtained. No additional clinical intervention, patient contact, or prospective data collection was involved. The requirement for informed consent was waived by the Ethics Committee because this study used pre-existing medical records and involved no direct patient contact or intervention. The study was conducted in accordance with institutional requirements for retrospective medical record-based research and the principles of the Declaration of Helsinki.

### Study area and population

This research was performed in Yixing City, a place in the southern area of Jiangsu Province in eastern China. Yixing spans around 1,997 square kilometers, with a permanent population of nearly 1.28 million by the end of 2024. Yixing People’s Hospital is the largest tertiary general hospital in the region and serves as the primary referral center for gastroscopic examinations across the city and surrounding areas. Its gastroenterology department performs a high volume of endoscopic procedures each year. We retrospectively identified all unique individuals who underwent at least one upper gastrointestinal endoscopy at the gastroenterology department of Yixing People’s Hospital between January 1, 2024 and December 31, 2025. For individuals who underwent multiple gastroscopic examinations during the study period, only the earliest examination within this period was retained, and all subsequent examinations were excluded before outcome classification and daily aggregation. Participants were not restricted by age, sex, or indication for endoscopy. This inclusive enrollment strategy was adopted to ensure the representativeness of the study population and to capture the full spectrum of gastroscopic findings during the study period.

### Data collection

After ethics approval was granted, we extracted five variables from the electronic medical record system, including sex, age, date of gastroscopic examination, endoscopic diagnosis, and detailed endoscopic description. None of these variables had missing values.

In this study, EGML was defined as an endoscopically visible erosive lesion involving the gastric mucosa, as documented in the gastroscopy report. This definition was based on endoscopic findings rather than pathological or etiological classification. Because the retrospective reports did not consistently include information on symptom duration, previous endoscopic findings, histopathology, medication exposure, or *Helicobacter pylori* status, EGML was not further classified into acute or chronic subtypes.

EGML were identified using a two-step procedure consisting of a predefined text-screening algorithm followed by gastroenterologist review. In the first step, the algorithm was applied to both the endoscopic diagnosis and the detailed endoscopic description. Before screening, the report text was standardized by harmonizing synonymous expressions, punctuation, and spacing. The algorithm first extracted report segments referring to gastric anatomical sites, including the cardia, gastric fundus, gastric body, gastric angle, gastric antrum, pylorus, remnant stomach, and anastomotic site when applicable. Report segments referring only to the esophagus or duodenum were not used to define EGML. A record was initially classified as EGML-positive when the gastric segment or endoscopic diagnosis contained erosion-related terms, including gastric erosion, erosive gastritis, verrucous gastritis, verrucous erosive lesions, acute gastric erosion, or acute gastric mucosal injury with documented gastric erosion.

To reduce false-positive classifications, the algorithm incorporated predefined exclusion rules for negated descriptions. Records were not classified as EGML-positive when erosion-related terms appeared only in negated contexts, such as no erosion, no obvious erosion, no gastric erosion, not accompanied by erosion, or erosion-negative descriptions. Isolated findings of congestion, edema, erythema, or bleeding without documented gastric erosion were not classified as EGML. Similarly, erosive lesions described only in the esophagus or duodenum were excluded.

In the second step, all algorithm-derived classifications were reviewed by four gastroenterologists, each with more than five years of clinical experience. The four physicians were divided into two pairs. For each record assigned to a pair, both physicians reviewed the algorithm-derived label together with the original endoscopy report. If the two physicians within a pair reached different conclusions, a more senior gastroenterologist made the final determination. The gastroenterologist-adjudicated classification was used as the final EGML outcome. After outcome classification, we aggregated the data to obtain the daily total number of gastroscopic examinations and the daily number of EGML cases throughout the study period.

We obtained daily concentrations of total PM_2.5_ and its five major chemical components from the TAP database (http://tapdata.org.cn). The TAP dataset provides gridded estimates at a spatial resolution of 10 km across China. These estimates were generated through a two-stage machine learning approach that integrates multiple data sources, including ground-based monitoring observations, simulations from the Community Multiscale Air Quality model, satellite-derived aerosol optical depth, meteorological reanalysis fields, and ancillary data on elevation, land use, and population density. In the first stage, a synthetic minority over-sampling technique was applied to improve the representation of high-pollution events, followed by random forest modeling. The second stage trained a separate random forest model on the residuals between modeled and observed concentrations to further refine prediction accuracy, with missing satellite retrievals imputed using decision tree-based algorithms. For the five components, total PM_2.5_ mass concentration was used as a constraint. Component fractions estimated by the air quality model were then calibrated against ground-level observations using an XGBoost algorithm to derive accurate conversion factors (18, 19). We downloaded gridded data covering Yixing City from December 1, 2023 to December 31, 2025. We then calculated the daily average concentrations of PM_2.5_ and its five components by averaging all grid points within the administrative boundary of Yixing City for each day. The extended period prior to the enrollment start date was included to allow for the calculation of lagged exposure windows.

In addition, daily meteorological and gaseous air pollutant data were collected from local environmental monitoring stations in Yixing over the same period. Daily meteorological indicators consisted of average temperature (°C), relative humidity (%), and wind speed (m/s). Gaseous air pollutants included daily mean concentrations of SO_2_ (μg/m^3^), NO_2_ (μg/m^3^), and CO (mg/m^3^), as well as O_3_ (μg/m^3^), which was expressed as the daily maximum 8-h moving average concentration.

### Statistical analysis

To assess the performance of the text-screening algorithm, the gastroenterologist-adjudicated classification was used as the reference standard. We constructed a two-by-two contingency table comparing the algorithm-derived classification with the final adjudicated EGML outcome. Sensitivity, specificity, positive predictive value, negative predictive value, accuracy, and Cohen’s kappa coefficient were calculated to evaluate the performance and agreement of the text-screening algorithm.

We adopted a time-series analytical framework to explore the relationships between daily levels of PM_2.5_ along with its five chemical constituents and the daily number of EGML cases. Generalized additive models (GAMs) with a quasi-Poisson distribution were utilized to account for overdispersion in the daily count data (20). The natural logarithm of the daily total number of gastroscopic examinations was included as an offset term, so that the model could effectively estimate the daily incidence rate of EGML rather than the absolute count (21). The association strength was expressed as the incidence rate ratio (IRR) with its 95% confidence interval (CI) per interquartile range (IQR) increase in pollutant concentration.

To capture potentially delayed health effects, we constructed cumulative moving average exposure metrics across 15 lag windows, ranging from lag 0–1 days to lag 0–15 days. Specifically, lag 0–1 days represented the average pollutant concentration on the gastroscopy day and the previous day, while lag 0–2 days indicated the mean concentration covering the gastroscopy day and the preceding two days, and this definition was extended sequentially up to lag 0–15 days. For each pollutant, a separate model was fitted for each lag window. The optimal exposure window was then identified as the one yielding the lowest generalized cross-validation (GCV) score (22). To mitigate the multiple comparison bias caused by evaluating 15 lag windows for each pollutant, the Benjamini-Hochberg false discovery rate (FDR) method was adopted for correction separately per pollutant (23).

Several covariates were adjusted for in all models. A thin plate regression spline for a continuous time variable was included to control for long-term trends and unmeasured slowly varying confounders, with the basis dimension set to 14 (K = 14, approximately seven per year of the two-year study period) (22). Season (cold or warm) and public holiday (yes or no) were also included as categorical covariates. The cold season was specified as January–March and October–December, with the warm season spanning April through September (24). Moreover, thin plate regression splines with a basis dimension of four (K = 4) were applied to the cumulative moving averages of daily average temperature, relative humidity, and wind speed at the corresponding lag window, thereby adjusting for the potential nonlinear effects of weather conditions (25).

We further conducted several sensitivity analyses. First, two-pollutant models were constructed by additionally adjusting for each of the four gaseous air pollutants (SO_2_, NO_2_, CO, and O_3_), using their cumulative moving average concentrations at the same lag window. This approach aimed to assess whether the observed associations were independent of co-existing gaseous pollutants. Second, we varied the basis dimension for the long-term time trend spline (k = 10, 12, 14, 16, and 18) to examine whether the results were sensitive to the flexibility of temporal adjustment. Third, we similarly varied the basis dimension for the meteorological splines (k = 3, 4, 5, 6, and 7) to test the sensitivity of results to the degree of control for meteorological factors.

Subgroup analyses were further stratified by sex (male vs. female), age group (<60 years vs. ≥ 60 years), and season (cold vs. warm season). In the sex-stratified and age-stratified analyses, the daily count of EGML cases and the corresponding daily total number of examinations within each subgroup were used as the outcome and offset, respectively. In the season-stratified analyses, the dataset was restricted to days within the cold or warm season, and the season indicator was removed from the model since it was no longer needed. To formally test whether the effect estimates differed between subgroups, we calculated a Z statistic using the formula: $Z=|\beta_{1}-\beta_{2}|/\sqrt{S{E_{1}}^{2}+S{E_{2}}^{2}}$ , where $\beta_{1}$ and $\beta_{2}$ are the regression coefficients from the two subgroup models, and $SE_{1}$ and $SE_{2}$ are their corresponding standard errors (26).

We further conducted an attributable burden analysis to estimate the number of EGML cases that could potentially be prevented under hypothetical pollution reduction scenarios. The attributable fraction (AF) for each day was calculated using the following formula: $\mathrm{AF}=1-\exp(-\beta \times \Delta_{x})$ , where β is the regression coefficient for the pollutant, and $\Delta_{x}$ represents the difference between the observed pollutant concentration and a counterfactual reference level. For days on which the observed concentration was below the counterfactual level, $\Delta_{x}$ was set to zero. Three hypothetical reduction scenarios were considered, assuming 25, 50, and 75% reductions from the observed mean concentration of each pollutant, respectively. The preventable number of cases under each scenario was then derived by multiplying the corresponding AF by the total number of EGML cases observed during the study period (27). Additionally, we quantified the relative contribution of each PM_2.5_ component to the overall health effect by computing a weighted effect size metric, defined as the product of (IRR-1) and the IQR for each component. The proportion of each component was then calculated as the ratio of its weighted effect size to the sum across all five components. All analyses were performed with R software version 4.5.2. A two-tailed *p*-value below 0.05 was regarded as statistically significant.

## Results

### Characteristics of the study population

During the period from January 1, 2024 to December 31, 2025, a total of 60,789 individuals underwent gastroscopic examination at Yixing People’s Hospital and were included in the present study. Of these participants, 28,599 (47.05%) were male, and the mean age was 55.09 ± 14.40 years. A total of 36,454 participants (59.97%) were younger than 60 years, 28,787 (47.36%) were examined in 2024, and 29,055 (47.80%) were examined during the cold season. Within the study period, 14,325 individuals were classified as having EGML. Among these cases, 7,970 (55.64%) were male, with a mean age of 57.85 ± 12.21 years; 7,738 (54.02%) were younger than 60 years, 6,720 (46.91%) were examined in 2024, and 6,970 (48.66%) were examined during the cold season (Table 1).

**Table 1:** Characteristics of study participants.

Characteristic	All participants undergoing gastroscopy (*N* = 60,789)	Participants with EGML (*N* = 14,325)
Sex, *n* (%)		
Male	28,599 (47.05)	7,970 (55.64)
Female	32,190 (52.95)	6,355 (44.36)
Age (years), mean ± SD	55.09 ± 14.40	57.85 ± 12.21
Age group, *n* (%)		
<60 years old	36,454 (59.97)	7,738 (54.02)
≥60 years old	24,335 (40.03)	6,587 (45.98)
Year, n (%)		
2024	28,787 (47.36)	6,720 (46.91)
2025	32,002 (52.64)	7,605 (53.09)
Season, *n* (%)		
Cold season	29,055 (47.80)	6,970 (48.66)
Warm season	31,734 (52.20)	7,355 (51.34)

EGML, erosive gastric mucosal lesions; SD, standard deviation.

### Performance of the text-screening algorithm for EGML identification

Using the gastroenterologist-adjudicated classification as the reference standard, the predefined text-screening algorithm showed good performance in identifying EGML. The sensitivity, specificity, positive predictive value, negative predictive value, and overall accuracy were 93.8, 96.8, 90.2, 98.1, and 96.1%, respectively. The Cohen’s kappa coefficient was 0.894, indicating good agreement between the algorithm-derived classification and the final gastroenterologist-adjudicated outcome (Supplementary Table S1).

### Characteristics of environmental factors

From December 1, 2023 to December 31, 2025, the median (25th percentile, 75th percentile) concentrations of PM_2.5_ and its five components in Yixing City were 28.61 μg/m^3^ (17.09, 45.04) for PM_2.5_, 4.68 μg/m^3^ (2.80, 7.75) for ${\mathrm{SO}}_{4}^{2-}$ , 5.65 μg/m^3^ (2.58, 10.14) for ${\mathrm{NO}}_{3}^{-}$ , 3.81 μg/m^3^ (1.71, 6.63) for ${\mathrm{NH}}_{4}^{+}$ , 6.05 μg/m^3^ (3.66, 9.49) for OM, and 1.13 μg/m^3^ (0.72, 1.72) for BC (Table 2).

**Table 2:** Characteristics of environmental factors during the study period.

Variable	Minimum	25th percentile	Median	Mean	75th percentile	Maximum
PM_2.5_ (μg/m^3^)	2.71	17.09	28.61	34.77	45.04	154.08
${\mathrm{SO}}_{4}^{2-}$ (μg/m^3^)	0.50	2.80	4.68	6.04	7.75	31.79
${\mathrm{NO}}_{3}^{-}$ (μg/m^3^)	0.23	2.58	5.65	7.64	10.14	43.64
${\mathrm{NH}}_{4}^{+}$ (μg/m^3^)	0.19	1.71	3.81	4.90	6.63	25.40
OM (μg/m^3^)	0.52	3.66	6.05	7.27	9.49	34.56
BC (μg/m^3^)	0.14	0.72	1.13	1.36	1.72	5.65
SO_2_ (μg/m^3^)	1.00	4.00	6.00	6.01	8.00	15.00
NO_2_ (μg/m^3^)	3.00	13.00	21.00	23.67	30.00	89.00
CO (mg/m^3^)	0.10	0.50	0.70	0.69	0.80	1.90
O_3_ (μg/m^3^)	3.00	49.00	72.00	76.64	101.00	200.00
Temperature (°C)	−5.40	8.10	18.30	17.34	26.20	33.80
Relative humidity (%)	27.00	67.00	76.00	74.34	84.00	100.00
Wind speed (m/s)	0.20	1.80	2.45	2.65	3.40	8.20

**Figure 1:**
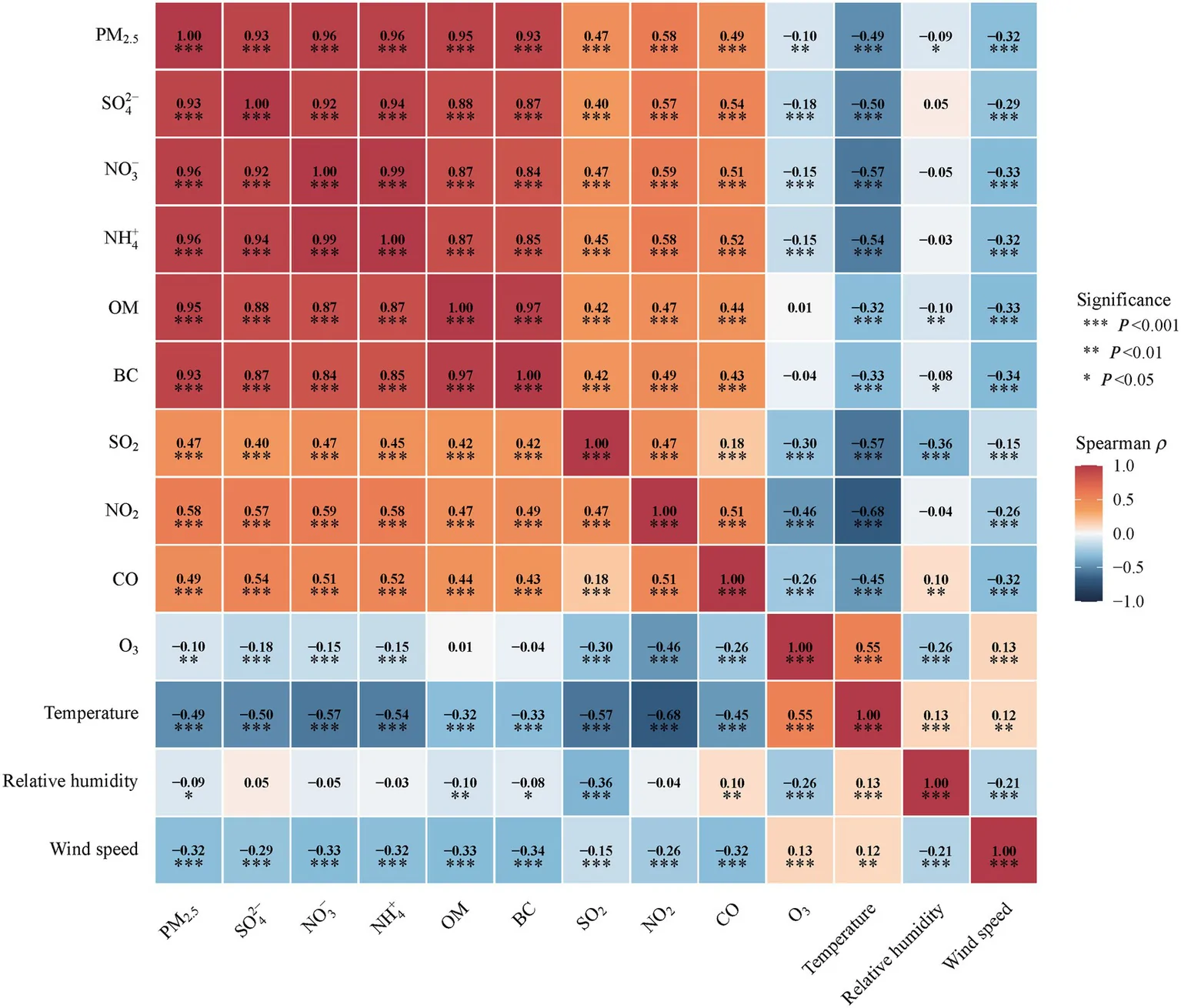
The Spearman correlation coefficients of environmental factors during the study period.

The Spearman correlation analyses revealed that PM_2.5_ and its five components were strongly and positively correlated with each other during the study period (all *p* < 0.001). These pollutants also showed moderate positive correlations with SO_2_, NO_2_, and CO (all *p* < 0.001), while PM_2.5_, ${\mathrm{SO}}_{4}^{2-}$ , ${\mathrm{NO}}_{3}^{-}$ , and ${\mathrm{NH}}_{4}^{+}$ were weakly and negatively correlated with O_3_ (all *p* < 0.01). In addition, PM_2.5_ and its five components were negatively correlated with temperature and wind speed to varying extents (all *p* < 0.001), and PM_2.5_, OM, and BC were weakly and negatively correlated with relative humidity (all *p* < 0.05) (Figure 1).

### Associations between PM_2.5_ and its five components and EGML

**Figure 2:**
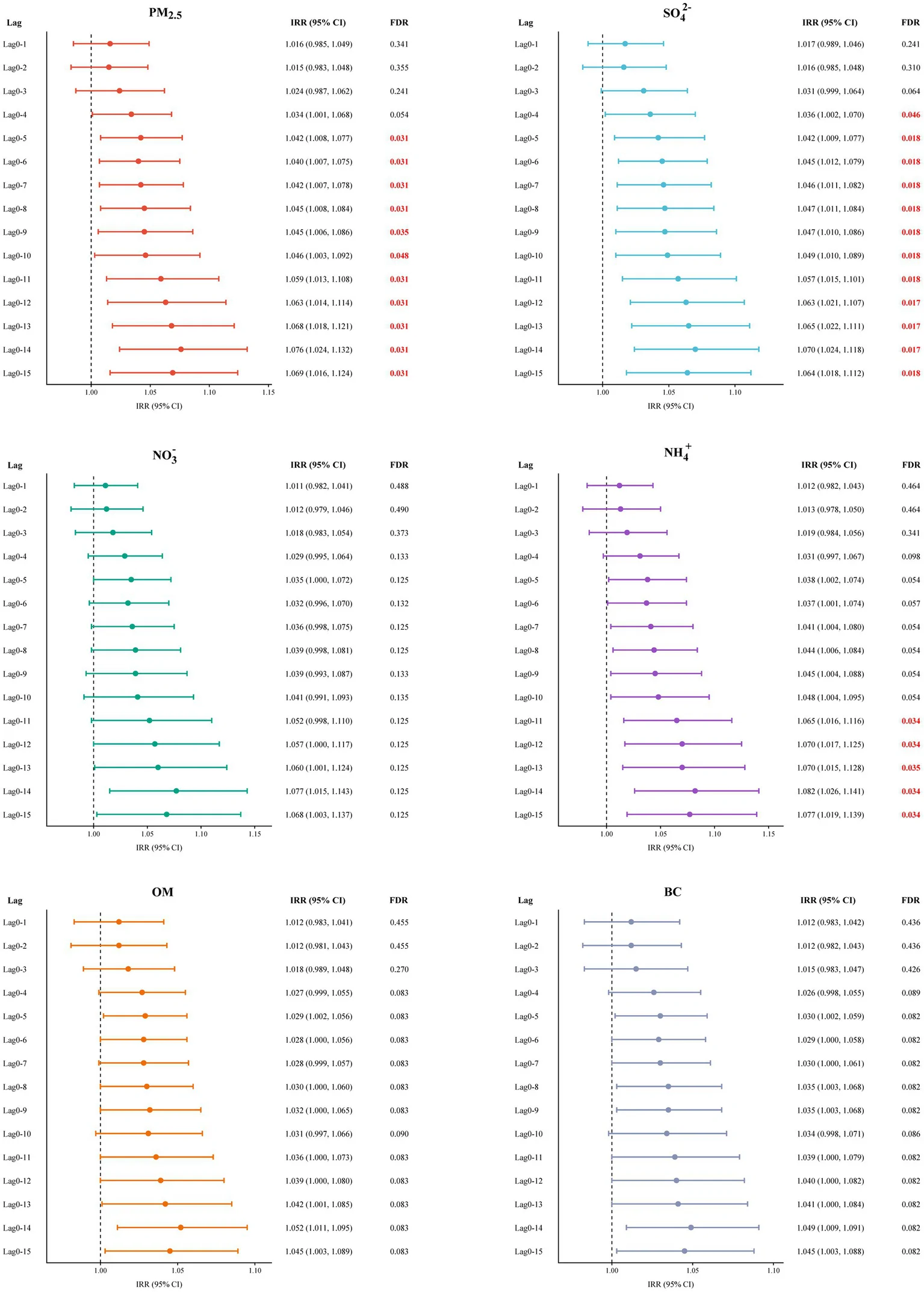
Associations between per interquartile range increase in PM_2.5_ and its components and erosive gastric mucosal lesions across different exposure windows (incidence rate ratio with 95% confidence interval).

PM_2.5_ was significantly and positively associated with EGML from lag 0–4 days to lag 0–15 days, and the positive associations remained significant from lag 0–5 days to lag 0–15 days after FDR correction. ${\mathrm{SO}}_{4}^{2-}$ showed significant positive associations with EGML from lag 0–4 days to lag 0–15 days, which persisted from lag 0–4 days to lag 0–15 days after FDR correction. ${\mathrm{NO}}_{3}^{-}$ was significantly and positively associated with EGML from lag 0–12 days to lag 0–15 days, but these associations no longer remained significant after FDR correction. For ${\mathrm{NH}}_{4}^{+}$ , significant positive associations were observed from lag 0–5 days to lag 0–15 days, and after FDR correction, the associations remained significant from lag 0–11 days to lag 0–15 days. OM was significantly and positively associated with EGML from lag 0–5 days to lag 0–6 days, from lag 0–8 days to lag 0–9 days, and from lag 0–13 days to lag 0–15 days, although these associations were no longer significant after FDR correction. Similarly, BC showed significant positive associations with EGML at lag 0–5 days, from lag 0–8 days to lag 0–9 days, at lag 0–12 days, and from lag 0–14 days to lag 0–15 days, but none of these associations remained significant after FDR correction (Figure 2).

Based on the principle of minimizing the GCV score, the optimal exposure windows for PM_2.5_, ${\mathrm{SO}}_{4}^{2-}$ , ${\mathrm{NO}}_{3}^{-}$ , ${\mathrm{NH}}_{4}^{+}$ , OM, and BC were lag 0–14 days, lag 0–14 days, lag 0–2 days, lag 0–14 days, lag 0–2 days, and lag 0–2 days, respectively (Supplementary Table S2). At the optimal exposure windows, each IQR increase in PM_2.5_, ${\mathrm{SO}}_{4}^{2-}$ , ${\mathrm{NO}}_{3}^{-}$ , ${\mathrm{NH}}_{4}^{+}$ , OM, and BC was associated with increases in EGML risk of 7.6% (IRR: 1.076; 95% CI: 1.024, 1.132), 7.0% (IRR: 1.070; 95% CI: 1.024, 1.118), 1.2% (IRR: 1.012; 95% CI: 0.979, 1.046), 8.2% (IRR: 1.082; 95% CI: 1.026, 1.141), 1.2% (IRR: 1.012; 95% CI: 0.981, 1.043), and 1.2% (IRR: 1.012; 95% CI: 0.982, 1.043), respectively (Figure 2). The positive associations of PM_2.5_, ${\mathrm{SO}}_{4}^{2-}$ , and ${\mathrm{NH}}_{4}^{+}$ with EGML remained significant in two-pollutant models, as well as when the basis dimensions of the splines for the long-term trend or meteorological factors were varied (all *p* < 0.05) (Supplementary Tables S3–S5).

The positive link between PM_2.5_ and EGML still reached statistical significance in females and in individuals aged 60 years and older. The positive association between ${\mathrm{SO}}_{4}^{2-}$ and EGML was significant in females and across different age groups, whereas the positive association between ${\mathrm{NH}}_{4}^{+}$ and EGML remained significant in females and in those aged 60 years and older (all *p* < 0.05) (Table 3). Moreover, gender significantly modified the effects of PM_2.5_ and ${\mathrm{NH}}_{4}^{+}$ on EGML (both *p* < 0.05) (Supplementary Table S6).

**Table 3:** Subgroup analyses of associations between per interquartile range increase in PM_2.5_ and its components and erosive gastric mucosal lesions (incidence rate ratio with 95% confidence interval).

Group	PM_2.5_	${\mathrm{SO}}_{4}^{2-}$	${\mathrm{NO}}_{3}^{-}$	${\mathrm{NH}}_{4}^{+}$	OM	BC
Overall	1.076 (1.024, 1.132)	1.070 (1.024, 1.118)	1.012 (0.979, 1.046)	1.082 (1.026, 1.141)	1.012 (0.981, 1.043)	1.012 (0.982, 1.043)
Gender						
Male	1.026 (0.967, 1.089)	1.040 (0.987, 1.095)	1.006 (0.970, 1.043)	1.032 (0.969, 1.098)	1.008 (0.975, 1.041)	1.006 (0.974, 1.038)
Female	1.133 (1.060, 1.210)	1.107 (1.046, 1.173)	1.029 (0.985, 1.074)	1.141 (1.064, 1.223)	1.028 (0.988, 1.069)	1.035 (0.997, 1.075)
Age group						
<60 years old	1.057 (0.989, 1.129)	1.063 (1.003, 1.126)	1.037 (0.995, 1.080)	1.071 (0.999, 1.148)	1.028 (0.991, 1.066)	1.028 (0.992, 1.065)
≥60 years old	1.076 (1.009, 1.146)	1.061 (1.004, 1.121)	0.993 (0.951, 1.038)	1.073 (1.003, 1.148)	1.006 (0.966, 1.047)	1.004 (0.965, 1.045)
Season						
Cold	1.075 (0.989, 1.170)	1.039 (0.969, 1.114)	1.009 (0.962, 1.058)	1.056 (0.974, 1.144)	1.010 (0.969, 1.054)	1.013 (0.965, 1.064)
Warm	1.011 (0.955, 1.070)	1.025 (0.971, 1.081)	1.003 (0.960, 1.047)	1.032 (0.977, 1.090)	1.004 (0.973, 1.037)	1.001 (0.972, 1.032)

**Figure 3:**
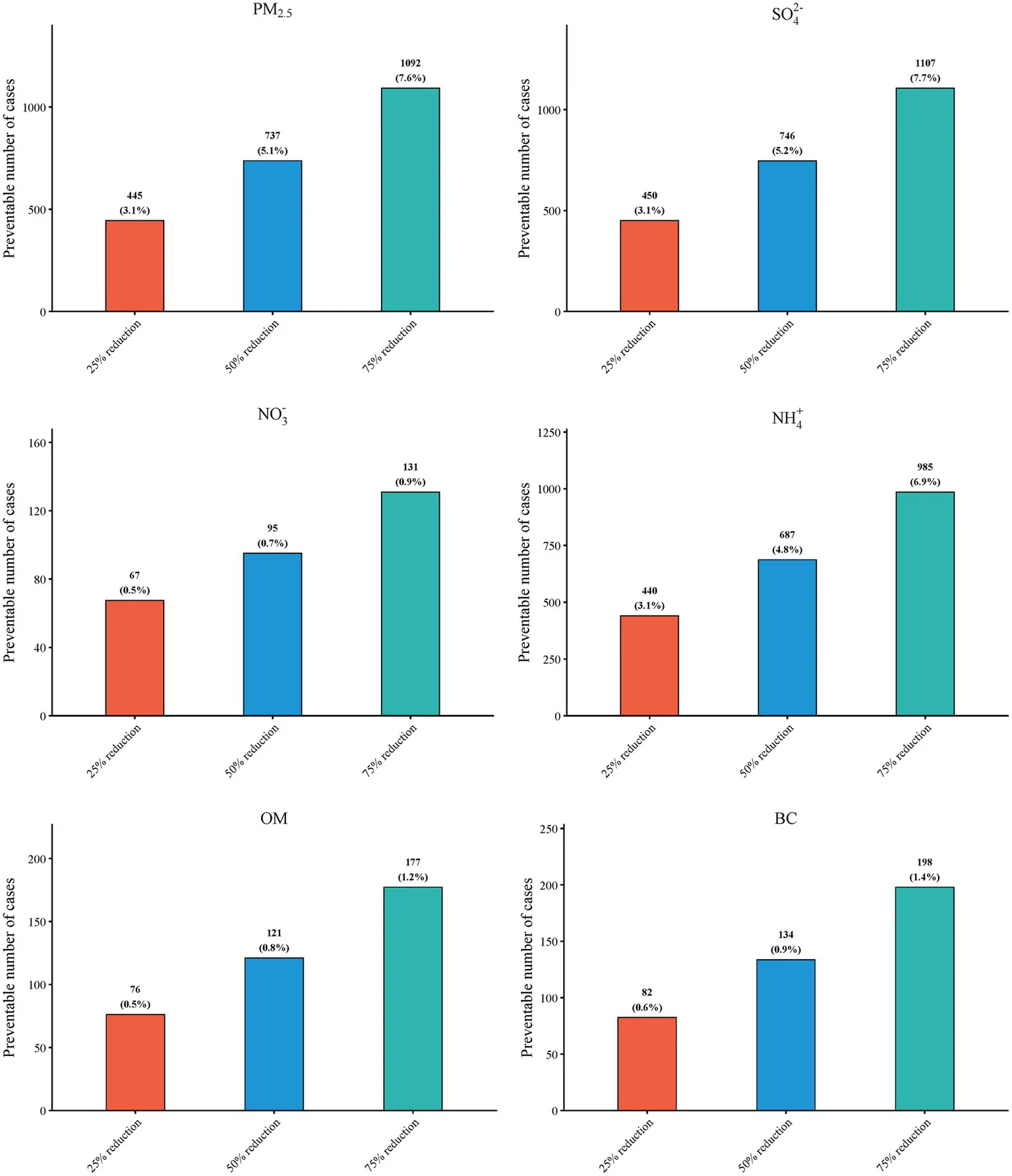
Preventable number of cases with erosive gastric mucosal lesions under different emission reduction scenarios.

**Figure 4:**
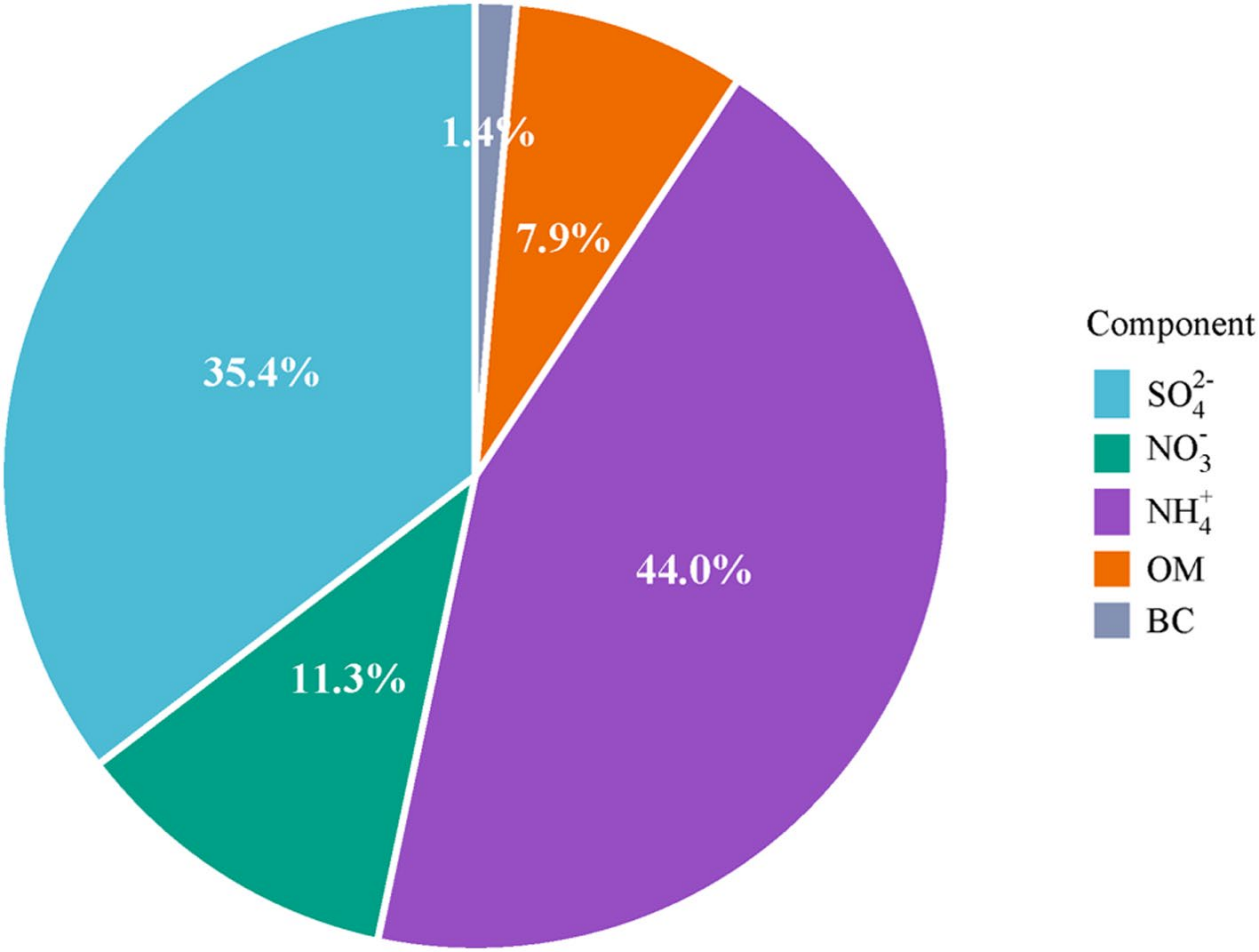
Contribution of different PM_2.5_ components.

Under hypothetical reductions of 25, 50, and 75% in PM_2.5_, 445 (3.1%), 737 (5.1%), and 1,092 (7.6%) EGML cases could be prevented, respectively. For ${\mathrm{SO}}_{4}^{2-}$ , the corresponding numbers were 450 (3.1%), 746 (5.2%), and 1,107 (7.7%) cases, and for ${\mathrm{NO}}_{3}^{-}$ , they were 67 (0.5%), 95 (0.7%), and 131 (0.9%) cases. Reductions of 25, 50, and 75% in ${\mathrm{NH}}_{4}^{+}$ were estimated to prevent 440 (3.1%), 687 (4.8%), and 985 (6.9%) cases, respectively. The corresponding numbers for OM were 76 (0.5%), 121 (0.8%), and 177 (1.2%) cases, and for BC were 82 (0.6%), 134 (0.9%), and 198 (1.4%) cases (Figure 3). Regarding the contribution of each component to the overall effect of PM_2.5_ on EGML, ${\mathrm{SO}}_{4}^{2-}$ , ${\mathrm{NO}}_{3}^{-}$ , ${\mathrm{NH}}_{4}^{+}$ , OM, and BC accounted for 35.4, 11.3, 44.0, 7.9, and 1.4%, respectively (Figure 4).

## Discussion

In this time-series study involving 60,789 gastroscopy participants in eastern China, short-term PM_2.5_ exposure was positively correlated with EGML. When the five major chemical components were examined individually, ${\mathrm{SO}}_{4}^{2-}$ and ${\mathrm{NH}}_{4}^{+}$ showed the most robust positive associations, whereas ${\mathrm{NO}}_{3}^{-}$ , OM, and BC were not significantly associated with EGML after correction for multiple testing. The attributable burden analysis further revealed that reducing ambient PM_2.5_ and its chemical constituents could avert new EGML cases to varying extents. These findings support further investigation of secondary inorganic aerosols in relation to gastric mucosal injury. To our knowledge, this is one of the first studies to correlate component-specific PM_2.5_ exposure with EGML in a general population, providing preliminary component-specific evidence relevant to air quality management and gastric disease prevention. Consistent with our finding that short-term PM_2.5_ exposure was positively associated with EGML, a recent case-crossover study in Yancheng, China reported that short-term exposure to PM_2.5_ increased gastrointestinal cancer mortality (28). However, that study focused on fatal gastrointestinal cancers and identified older males as the most vulnerable subgroup, whereas our study assessed endoscopically detected EGML and found stronger associations in females and older adults, thereby extending the evidence to gastric mucosal lesions and PM_2.5_ component-specific associations.

Previous component-specific epidemiological studies have reported that ${\mathrm{SO}}_{4}^{2-}$ and ${\mathrm{NH}}_{4}^{+}$ were associated with increased risks of respiratory and cardiovascular outcomes, including asthma (29) and myocardial infarction (30). Evidence from a retrospective cohort study also suggested positive associations of ${\mathrm{SO}}_{4}^{2-}$ and ${\mathrm{NH}}_{4}^{+}$ with colorectal cancer (31). Direct evidence linking ${\mathrm{SO}}_{4}^{2-}$ and ${\mathrm{NH}}_{4}^{+}$ to EGML remains scarce, yet such associations possess plausible biological mechanisms. ${\mathrm{SO}}_{4}^{2-}$ and ${\mathrm{NH}}_{4}^{+}$ are major constituents of secondary inorganic aerosols, which are highly water-soluble and may be deposited in the upper airway, cleared by mucociliary transport, and subsequently swallowed into the gastrointestinal tract, leading to potential contact with the gastric mucosa (32, 33). Although epidemiological studies specifically focusing on gastric mucosal lesions are lacking, studies of other gastrointestinal outcomes, such as ulcerative colitis and gastrointestinal cancer, suggest that particulate air pollution and its secondary inorganic components may contribute to gastrointestinal injury through inflammatory and oxidative pathways (31, 34, 35). Further studies combining component-specific exposure assessment with endoscopic outcomes and biomarkers of gastric inflammation, oxidative stress, and mucosal barrier dysfunction are warranted.

We evaluated a series of cumulative moving average exposure windows, which allowed us to capture both immediate effects and short-term cumulative effects of PM_2.5_ at the same time. We applied the minimum GCV criterion to select the optimal window for each pollutant. The results showed that the optimal windows for PM_2.5_, ${\mathrm{SO}}_{4}^{2-}$ , and ${\mathrm{NH}}_{4}^{+}$ all fell at lag 0–14 days, suggesting that the association was likely to be a short-term cumulative process. This observation seems reasonable, as gastric mucosal erosion typically develops after aggressive factors such as gastric acid, bile reflux, alcohol, medications, and inflammation outweigh mucosal defense over a period of time (36, 37). An exposure window of around two weeks may therefore reflect the process by which particulate matter induces systemic inflammation, affects mucosal microcirculation, impairs epithelial repair, or amplifies pre-existing mucosal vulnerability (38). This time scale is consistent with evidence that PM_2.5_-related systemic inflammatory and oxidative responses may occur within hours to several days and may be sustained by repeated exposure (39, 40). Therefore, the lag 0–14 days pattern does not necessarily imply immediate direct erosion by particles in the gastric lumen, but may also reflect indirect inflammatory injury, microcirculatory impairment, or delayed mucosal repair before lesions become endoscopically apparent. In contrast, ${\mathrm{NO}}_{3}^{-}$ , OM, and BC showed smaller estimates with wider confidence intervals, and none remained significant after FDR correction. Their null findings cannot be explained by low concentration variability alone. ${\mathrm{NO}}_{3}^{-}$ had the largest IQR among the five components at 7.56 μg/m^3^, while the IQR of OM was 5.83 μg/m^3^, compared with 4.95 μg/m^3^ for ${\mathrm{SO}}_{4}^{2-}$ and 4.92 μg/m^3^ for ${\mathrm{NH}}_{4}^{+}$ . In the overall evaluation of the TAP component data, the correlation coefficients between predicted and observed concentrations were 0.70 for ${\mathrm{SO}}_{4}^{2-}$ , 0.75 for ${\mathrm{NO}}_{3}^{-}$ , 0.75 for ${\mathrm{NH}}_{4}^{+}$ , 0.72 for OM, and 0.64 for BC; the corresponding normalized mean biases were −15.57, 6.57, 7.19, −26.08%, and −17.66% (19). The weaker performance for OM and BC may have increased exposure error and attenuated their estimates. This explanation is less applicable to ${\mathrm{NO}}_{3}^{-}$ , which had high variability and relatively good prediction performance. Correlations among components may also limit component-specific attribution, but they do not by themselves explain null results from separate single-component models. The findings for ${\mathrm{NO}}_{3}^{-}$ , OM, and BC may therefore reflect smaller true associations, exposure error, or statistical uncertainty, and should not be interpreted as evidence of no effect.

An important issue is that EGML in this study represents an endoscopic lesion phenotype rather than a temporally defined clinical diagnosis. Although erosive gastritis can be clinically and pathologically classified into acute and chronic forms, routine endoscopy reports usually document the presence of mucosal erosions but do not reliably indicate when the lesions developed. Therefore, our findings should not be interpreted as evidence that short-term PM_2.5_ exposure exclusively induces newly developed acute erosions. Instead, short-term exposure may be associated with the occurrence, aggravation, delayed repair, symptom presentation, or endoscopic detection of gastric erosive lesions.

Sensitivity analyses further strengthened the robustness of the main results. Gaseous pollutants including SO_2_, NO_2_, CO, and O_3_ often co-occur with particulate pollution. After adjustment for these gaseous pollutants, the positive associations of PM_2.5_, ${\mathrm{SO}}_{4}^{2-}$ , and ${\mathrm{NH}}_{4}^{+}$ with EGML remained significant, suggesting that the observed associations were not fully explained by gaseous pollutants. The findings also remained stable when the basis dimensions of the long-term trend spline and the meteorological splines were varied. Since effect estimates in time-series studies can be influenced by the handling of long-term trends and meteorological factors, these sensitivity checks were necessary (41). The consistency across different model specifications indicated that our findings were unlikely to be driven by any single modeling choice. Subgroup analyses showed that the positive associations were more pronounced in females and older adults, with statistically significant effect modification by sex for PM_2.5_ and ${\mathrm{NH}}_{4}^{+}$ . Several factors may account for this pattern. Females tend to have lower basal gastric acid secretion and depend more on mucosal defense mechanisms, so the physiological balance may be disrupted more easily following additional external oxidative stress (42). Older adults often present with reduced mucosal regeneration capacity, a higher prevalence of *Helicobacter pylori* colonization, and more frequent use of nonsteroidal anti-inflammatory drugs, all of which may amplify the impact of environmental exposure (43). Although the seasonal stratification did not reveal marked heterogeneity, the positive associations persisted in both cold and warm seasons, further ruling out confounding solely by seasonal behavioral patterns.

Several underlying mechanisms may account for the link between PM_2.5_ exposure and EGML. First, inhaled fine particles may trigger systemic inflammation and oxidative stress, and the subsequent rise in circulating cytokines and reactive oxygen species may compromise the mucus bicarbonate barrier, disturb mucosal microcirculation, and weaken epithelial repair (44–46). Second, a direct luminal pathway is biologically plausible because a fraction of inhaled particles deposited in the airway can be transported to the oropharynx by mucociliary clearance, and subsequently swallowed into the gastric lumen (47). However, the quantity, concentration, and chemical form of particles reaching the stomach under ambient exposure remain difficult to determine. Therefore, direct contact should be regarded as a plausible but unproven contributor rather than the sole explanation. Ingested particulates may disrupt tight junctions, attenuate the mucus layer, and trigger local inflammatory infiltration, thereby compromising mucosal integrity (48). Third, PM_2.5_ exposure is likely to alter gut microbiota composition, and such dysbiosis may influence gastric mucosal defense through microbial metabolites, immune regulation, and gut-stomach interactions (49, 50). Thus, the observed short-term association is compatible with combined systemic, swallowed-particle, and microbiota-mediated pathways, although their relative contributions cannot be distinguished in this study.

This study has several strengths. First, the outcome was identified through endoscopic findings, which is more objective. A two-step ascertainment procedure combining text screening with independent review by gastroenterologists improved the efficiency of handling a large sample while preserving clinical validity. Second, the time-series design inherently controls for individual characteristics that remain relatively stable over short periods, since comparisons were made across different days within the same city. Third, exposure estimates were drawn from high-resolution gridded data covering PM_2.5_ and its five chemical components, and multiple sensitivity and subgroup analyses together supported the robustness of our findings. Fourth, including the daily total number of gastroscopic examinations as an offset minimized the influence of day-to-day fluctuations in medical service volume. Finally, the attributable burden analysis translated statistical associations into preventable case numbers under realistic reduction scenarios, which offers more actionable information for policymakers than effect estimates alone.

Some limitations should also be acknowledged. First, this is a single-center study, and the study population consisted of individuals who underwent gastroscopy, so the outcome reflects the detection of EGML among examinees rather than the true incidence in the general community. In addition, because the outcome was derived from retrospective endoscopy reports, we could not distinguish acute erosive gastritis from chronic erosive gastritis or determine the exact onset time of each lesion. Some lesions detected during the study period may therefore have developed before the examined exposure window. Second, we calculated city-level daily mean pollutant concentrations from 10 km × 10 km TAP grids. However, the limited number of grids covering Yixing and city-level averaging may mask within-city spatial heterogeneity and fail to capture population-weighted, indoor, occupational, or mobility-related exposures. Such exposure misclassification would likely attenuate associations if non-differential, although the direction of bias could be uncertain if examinees were disproportionately drawn from areas with pollution trends different from the city-level average. Third, we were unable to obtain daily population-level information on several important gastric risk factors, including *Helicobacter pylori* infection, alcohol use, smoking, psychological stress, and medication use, so residual confounding cannot be ruled out entirely. If factors such as alcohol use, smoking, or nonsteroidal anti-inflammatory drug use varied seasonally or temporally in parallel with air pollution levels, positive confounding may have occurred and the observed associations could have been partly overestimated. Conversely, relatively stable factors, such as chronic *Helicobacter pylori* infection or habitual smoking, are less likely to explain short-term variations in EGML detection, although they may modify individual susceptibility. Although we adjusted for long-term trends, season, public holidays, and meteorological conditions, residual confounding by unmeasured time-varying behaviors or clinical factors remains possible. Finally, because systemic inflammatory biomarkers, gastric mucosal biomarkers, microbiota data, and estimates of swallowed particle dose were unavailable, we could not determine the relative contributions of direct luminal contact, systemic inflammation, and gut-stomach-axis pathways. Future research could extend validation to more regions, incorporate individual-level exposure assessment, collect more detailed gastric risk factor information, and further examine biomarkers of inflammation, oxidative stress, mucosal barrier function, and microbial changes.

## Conclusion

We observed that short-term exposure to PM_2.5_ correlates with an increased risk of endoscopically identified EGML. Secondary inorganic components, particularly ${\mathrm{SO}}_{4}^{2-}$ and ${\mathrm{NH}}_{4}^{+}$ , showed the strongest evidence of positive associations. These findings extend current epidemiological evidence on air pollution and gastric health, and they point to the potential value of source-oriented air quality management, especially the control of secondary inorganic aerosols, for reducing the environmental burden of erosive gastric disease.

## Data Availability

The original contributions presented in the study are included in the article/Supplementary material, further inquiries can be directed to the corresponding author/s.
