# Supplementary material for: Component-specific effects of PM2.5 on endoscopically identified erosive gastric mucosal lesions: a time-series study in Yixing, China
Source: Front Public Health. 2026 Aug 11;14:1891236. doi: 10.3389/fpubh.2026.1891236 (PMC13503573; doi:10.3389/fpubh.2026.1891236)
Supplement: Supplementary file 1 [file Supplementary_file_1.docx]

**Table S1. Performance of the predefined text-screening algorithm for identifying erosive gastric mucosal lesions.**

| Metric | Value |
| --- | --- |
| Sensitivity | 93.8% |
| Specificity | 96.8% |
| Positive predictive value | 90.2% |
| Negative predictive value | 98.1% |
| Accuracy | 96.1% |
| kappa | 0.894 |

**Table S2. Generalized cross-validation scores for models with different exposure windows.**

| Exposure window | PM_2.5_ | $\mathrm{SO}_{4}^{2-}$ | $\mathrm{NO}_{3}^{-}$ | $\mathrm{NH}_{4}^{+}$ | OM | BC |
| --- | --- | --- | --- | --- | --- | --- |
| Lag 0-1 days | 1.1640 | 1.1635 | 1.1645 | 1.1645 | 1.1644 | 1.1644 |
| Lag 0-2 days | 1.1630 | 1.1629 | **1.1634** | 1.1634 | **1.1632** | **1.1632** |
| Lag 0-3 days | 1.1663 | 1.1697 | 1.1670 | 1.1670 | 1.1663 | 1.1673 |
| Lag 0-4 days | 1.1710 | 1.1705 | 1.1729 | 1.1724 | 1.1717 | 1.1722 |
| Lag 0-5 days | 1.1685 | 1.1680 | 1.1717 | 1.1707 | 1.1706 | 1.1709 |
| Lag 0-6 days | 1.1695 | 1.1670 | 1.1731 | 1.1715 | 1.1718 | 1.1724 |
| Lag 0-7 days | 1.1701 | 1.1681 | 1.1734 | 1.1714 | 1.1729 | 1.1730 |
| Lag 0-8 days | 1.1692 | 1.1678 | 1.1724 | 1.1701 | 1.1716 | 1.1712 |
| Lag 0-9 days | 1.1698 | 1.1678 | 1.1732 | 1.1706 | 1.1714 | 1.1708 |
| Lag 0-10 days | 1.1720 | 1.1691 | 1.1746 | 1.1718 | 1.1734 | 1.1734 |
| Lag 0-11 days | 1.1690 | 1.1660 | 1.1714 | 1.1681 | 1.1708 | 1.1711 |
| Lag 0-12 days | 1.1658 | 1.1639 | 1.1679 | 1.1651 | 1.1676 | 1.1678 |
| Lag 0-13 days | 1.1647 | 1.1619 | 1.1666 | 1.1636 | 1.1667 | 1.1672 |
| Lag 0-14 days | **1.1618** | **1.1600** | 1.1648 | **1.1611** | 1.1642 | 1.1653 |
| Lag 0-15 days | 1.1629 | 1.1611 | 1.1654 | 1.1622 | 1.1653 | 1.1659 |

**Table S3. Associations between PM_2.5_ and its components and endoscopically identified erosive gastric mucosal lesions in two-pollutant models (incidence rate ratio with 95% confidence interval).**

| Co-pollutant | PM_2.5_ | $\mathrm{SO}_{4}^{2-}$ | $\mathrm{NO}_{3}^{-}$ | $\mathrm{NH}_{4}^{+}$ | OM | BC |
| --- | --- | --- | --- | --- | --- | --- |
| SO_2_ | 1.106 (1.036, 1.180) | 1.088 (1.031, 1.148) | 1.021 (0.987, 1.057) | 1.104 (1.036, 1.176) | 1.021 (0.989, 1.054) | 1.024 (0.992, 1.058) |
| NO_2_ | 1.066 (1.007, 1.128) | 1.063 (1.012, 1.117) | 1.011 (0.976, 1.048) | 1.072 (1.011, 1.136) | 1.012 (0.979, 1.045) | 1.012 (0.979, 1.046) |
| CO | 1.060 (1.005, 1.118) | 1.051 (1.001, 1.104) | 1.012 (0.980, 1.045) | 1.065 (1.006, 1.127) | 1.012 (0.984, 1.042) | 1.015 (0.988, 1.044) |
| O_3_ | 1.076 (1.023, 1.133) | 1.070 (1.024, 1.119) | 1.013 (0.979, 1.048) | 1.084 (1.027, 1.144) | 1.013 (0.981, 1.046) | 1.023 (0.995, 1.052) |

**Table S4. Associations between PM_2.5_ and its components and endoscopically identified erosive gastric mucosal lesions across different degrees of freedom for the long-term trend (incidence rate ratio with 95% confidence interval).**

| Degree of freedom | PM_2.5_ | $\mathrm{SO}_{4}^{2-}$ | $\mathrm{NO}_{3}^{-}$ | $\mathrm{NH}_{4}^{+}$ | OM | BC |
| --- | --- | --- | --- | --- | --- | --- |
| 10 | 1.076 (1.024, 1.132) | 1.070 (1.024, 1.118) | 1.016 (0.983, 1.050) | 1.082 (1.026, 1.141) | 1.015 (0.985, 1.046) | 1.021 (0.993, 1.049) |
| 12 | 1.076 (1.024, 1.132) | 1.070 (1.024, 1.118) | 1.014 (0.981, 1.048) | 1.082 (1.026, 1.141) | 1.013 (0.983, 1.045) | 1.013 (0.983, 1.044) |
| 14 | 1.076 (1.024, 1.132) | 1.070 (1.024, 1.118) | 1.012 (0.979, 1.046) | 1.082 (1.026, 1.141) | 1.012 (0.981, 1.043) | 1.012 (0.982, 1.043) |
| 16 | 1.076 (1.024, 1.132) | 1.070 (1.024, 1.118) | 1.005 (0.971, 1.039) | 1.082 (1.026, 1.141) | 1.006 (0.975, 1.038) | 1.005 (0.974, 1.036) |
| 18 | 1.076 (1.023, 1.132) | 1.070 (1.024, 1.118) | 1.007 (0.974, 1.042) | 1.082 (1.026, 1.141) | 1.008 (0.977, 1.040) | 1.007 (0.976, 1.038) |

**Table S5. Associations between PM_2.5_ and its components and endoscopically identified erosive gastric mucosal lesions across different degrees of freedom for the meteorological factors (incidence rate ratio with 95% confidence interval).**

| Degree of freedom | PM_2.5_ | $\mathrm{SO}_{4}^{2-}$ | $\mathrm{NO}_{3}^{-}$ | $\mathrm{NH}_{4}^{+}$ | OM | BC |
| --- | --- | --- | --- | --- | --- | --- |
| 3 | 1.075 (1.023, 1.130) | 1.075 (1.029, 1.123) | 1.012 (0.979, 1.046) | 1.083 (1.027, 1.141) | 1.012 (0.981, 1.043) | 1.012 (0.982, 1.043) |
| 4 | 1.076 (1.024, 1.132) | 1.070 (1.024, 1.118) | 1.012 (0.979, 1.046) | 1.082 (1.026, 1.141) | 1.012 (0.981, 1.043) | 1.012 (0.982, 1.043) |
| 5 | 1.078 (1.026, 1.133) | 1.069 (1.023, 1.117) | 1.012 (0.979, 1.046) | 1.081 (1.025, 1.141) | 1.011 (0.981, 1.043) | 1.012 (0.982, 1.043) |
| 6 | 1.065 (1.011, 1.123) | 1.069 (1.022, 1.117) | 1.011 (0.978, 1.046) | 1.074 (1.016, 1.134) | 1.011 (0.981, 1.043) | 1.012 (0.982, 1.042) |
| 7 | 1.067 (1.012, 1.124) | 1.060 (1.011, 1.110) | 1.011 (0.978, 1.046) | 1.075 (1.018, 1.135) | 1.011 (0.981, 1.043) | 1.012 (0.981, 1.042) |

**Table S6. Effect modification of gender, age group, and season on the associations between PM_2.5_ and its components and endoscopically identified erosive gastric mucosal lesions.**

| Variable | PM_2.5_ | $\mathrm{SO}_{4}^{2-}$ | $\mathrm{NO}_{3}^{-}$ | $\mathrm{NH}_{4}^{+}$ | OM | BC |
| --- | --- | --- | --- | --- | --- | --- |
| Gender | **2.180** | 1.608 | 0.780 | **2.101** | 0.764 | 1.147 |
| Age group | 0.378 | 0.033 | 1.402 | 0.049 | 0.781 | 0.854 |
| Season | 0.711 | 0.097 | 0.074 | 0.098 | 0.095 | 0.269 |
